# Clostridium difficile Toxoid Vaccine Candidate Confers Broad Protection against a Range of Prevalent Circulating Strains in a Nonclinical Setting

**DOI:** 10.1128/IAI.00742-17

**Published:** 2018-05-22

**Authors:** Laurence Quemeneur, Nadine Petiot, Nadège Arnaud-Barbe, Catherine Hessler, Patricia J. Pietrobon, Patricia Londoño-Hayes

**Affiliations:** a Research & Development, Sanofi Pasteur, Marcy l'Etoile, France; b Research & Development, Sanofi Pasteur, Swiftwater, Pennsylvania, USA; University of Michigan—Ann Arbor

**Keywords:** Clostridium difficile, Clostridium difficile toxoid vaccine, protection, toxin-variant strains, efficacy

## Abstract

Clostridium difficile infection (CDI) is a leading cause of nosocomial and antibiotic-associated diarrhea. A vaccine, based on formalin-inactivated toxins A and B purified from anaerobic cultures of C. difficile strain VPI 10463 (toxinotype 0), has been in development for the prevention of symptomatic CDI. We evaluated the breadth of protection conferred by this C. difficile toxoid vaccine in cross-neutralization assessments using sera from vaccinated hamsters against a collection of 165 clinical isolates. Hamster antisera raised against the C. difficile toxoid vaccine neutralized the cytotoxic activity of culture supernatants from several toxinotype 0 strains and heterologous strains from 10 different toxinotypes. Further assessments performed with purified toxins confirmed that vaccine-elicited antibodies can neutralize both A and B toxins from a variety of toxinotypes. In the hamster challenge model, the vaccine conferred significant cross-protection against disease symptoms and death caused by heterologous C. difficile strains from the most common phylogenetic clades, including the most prevalent toxinotypes.

## INTRODUCTION

Clostridium difficile, a Gram-positive, spore-forming anaerobic bacterial pathogen, is a leading cause of nosocomial and antibiotic-associated diarrheal disease worldwide (1). The pathogen is responsible for 10% to 25% of antibiotic-associated diarrhea and for almost all cases of pseudomembranous colitis (2). A vaccine that protects against C. difficile infection (CDI) is needed given its increasing incidence, the substantial health care burden, and the limited treatment options (3, 4). CDI pathogenicity is mainly mediated by two exotoxins termed TcdA and TcdB (toxins A and B, respectively) (5–7), which makes them suitable targets for vaccine development; both toxins are monoglycosyl transferases, capable of causing cytoskeleton disorganization, via inactivation of Rho family GTPases. These toxins are responsible for the loss of epithelial barrier function, leading to increased intestinal permeability and fluid accumulation followed by the onset of diarrhea, a key characteristic feature of CDI (5–7).

TcdA and TcdB are encoded by a 19.6-kb chromosomal region termed the pathogenicity locus (PaLoc). C. difficile strain variants are commonly grouped by toxinotype, according to variations in the organization and sequence of their PaLoc compared to the reference strain, VPI 10463, in which the toxin genes were first sequenced and were designated toxinotype 0 (8, 9). A total of 34 different toxinotypes have been identified so far (9). The vast majority of pathogenic C. difficile strains express both TcdA and TcdB (8, 9) and are denoted phenotype A+B+. However, as a result of variations in the PaLoc, some prevalent pathogenic strains produce only TcdB (phenotype A−B+). In addition to expressing TcdA and TcdB, some epidemic strains produce a third toxin, C. difficile binary toxin (CDT) (10), and are denoted A+B+CDT+.

Molecular epidemiology studies conducted across several countries (North America and Europe [11–16], Latin America [17], and Asia [18]) over the last decade have identified seven prevalent toxinotypes (toxinotypes 0, III, IV, V, VIII, IX, and XII). Fluoroquinolone-resistant strains belonging to toxinotype III (A+B+CDT+ strains), also known as PCR ribotype (RT) 027 strains, have been identified as hypervirulent epidemic strains responsible for CDI outbreaks with high mortality (19). Toxinotype V/RT 078 (A+B+CDT+) strains are also hypervirulent strains associated with severe disease (20, 21). Toxinotype IV/RT 023 (A+B+CDT+) strains have emerged recently in various countries (20), and toxinotype VIII/RT 017 (A–B+CDT–) strains are highly prevalent in the Asia-Pacific region (18).

A C. difficile toxoid vaccine, based on formalin-inactivated toxins A and B purified from anaerobic cultures of C. difficile reference strain VPI 10463 (toxinotype 0), was shown to induce a robust dose-dependent anti-toxin A and B IgG response leading to protection in preclinical CDI models (22), with serum toxin-specific neutralizing antibody (Ab) titers correlating with protection (23). Phase I and II studies (24–26) have shown that the candidate vaccine has an acceptable safety profile and is immunogenic, with a robust immune response to both toxins observed in vaccinated healthy adults aged 18 to 55 years or ≥65 years, as well as in “at-risk” adults and elderly. The vaccine has recently undergone phase III assessment (ClinicalTrials registration no. NCT01887912).

In light of the evolving molecular epidemiology of CDI, it is important to evaluate the breadth of protection conferred by the candidate vaccine. With this aim, we assembled a collection of 165 clinical isolates and prototype strains of 11 different toxinotypes that are broadly representative of recent prevalent circulating strains in Europe, North America, Latin America, and the Asia-Pacific region. To ensure the representativeness of the collection, some of the isolates within each prevalent toxinotype group were further characterized by sequencing of both toxin genes and compared to the toxinotype 0 vaccine strain. We investigated whether polyclonal antibodies elicited by the vaccine could neutralize toxins secreted in culture by the whole collection of heterologous C. difficile isolates, and we purified toxin A or toxin B from the most prevalent toxinotypes. We also investigated whether the vaccine conferred cross-protection in the hamster model against lethal challenge with prototype variant strains from the most common phylogenetic clades, including the most prevalent toxinotypes (0, III, IV, V, and VIII).

(All or part of the information was presented at the 5th International Clostridium difficile Symposium, Bled, Slovenia, May 2015; at Nosocomial Infection Days, 7th edition, Lyon, France, December 2015; and at Vaccinology Symposium, Lyon, France, March 2017.)

## RESULTS

### C. difficile strains assessed.

The large collection of 165 C. difficile strains assessed, including 153 recent clinical isolates, was selected to be representative of the current molecular epidemiology worldwide (see the supplemental material). The geographical origins and molecular profiles of the isolates, including the toxinotypes, toxin production phenotypes, and ribotypes, are summarized in Table 1. Eleven different toxinotypes were represented, including the most prevalent toxinotypes from the most common phylogenetic clades, i.e., clades 0, III, IV, V, and VIII. More than 23 different RTs were represented. Interestingly, the toxinotype of two isolates collected in the Asia-Pacific region, RT 046 and RT 369, has yet to be defined. Twelve prototype strains across four toxinotypes were also included.

**TABLE 1:** Summary of C. difficile clinical isolates and prototype strains^*a*^

Toxinotype	Toxin production phenotype	PCR RT(s)	No. of isolates by geographical origin (*n* = 165)				Prototype strain(s)	No. of strains sequenced
			Europe^*b*^	USA	Argentina	Asia-Pacific^*c*^		
0	A+B+CDT−	087	2				VPI 10463	1
		012	3				**630**	1
		001	5	1			NCTC11204, NCTC11209	4
		002	5	2		5	NCTC12729	3
		014	5					
		020	4					1
		014, 020				5		2
		014, 020, 077	3					1
		106	2					2
		018	1			2		3
		053				1		
		Others	37	5	2	3		5
I	A+B+CDT−	ND^*d*^	1	0				1
III	A+B+CDT+	027	5	4			**IPP40348,** CDC13695, ATCC BAA-1870, R20291, CD196	11
		075	1					
		Others	1					
IV	A+B+CDT+	023	4 (**NK91**)					3
V	A+B+CDT+	078	3	2			**ATCC BAA-1875**	3
		079	1					
		122	1					
		126	4	0				
		078, 126	2					1
		ND	1					
VI	A+B+CDT+	127	2			2		1
VIII	A−B+CDT−	017	7	1	2	5	**ATCC 43598**	**4**
IX	A+B+CDT−	019	1					1
XII	A+B+CDT−	056				1		
		ND	1					1
Others		046				5		
		369				3		
Total			102	15	4	32	12	49

a Strains indicated in bold were used as the prototypes for *in vivo* cross-protection studies. RT, ribotype.

b The countries of Europe included Belgium, France, Germany, Ireland, Hungary, Italy, Netherlands, Poland, Spain, Switzerland, Sweden, Turkey, Greece, and the United Kingdom.

c The countries of Asia-Pacific included Japan, South Korea, Singapore, Taiwan, and Australia.

d ND, PCR ribotype unknown.

### Genome sequencing and phylogenetic analysis.

Whole-genome sequencing, which included genes encoding toxins A and B, was established for 49 of the C. difficile clinical isolates, representing eight different toxinotypes (Table 1). Repeats in the toxin A receptor-binding domain caused breaks in the *de novo* assembly and thus prevented full sequence determinations for the toxin A gene of 10 of these isolates. Pairwise sequence comparison at the amino acid level among strains within the same toxinotype revealed a high degree of sequence conservation (>98% sequence identity among strains of toxinotypes 0, III, and IV). Similar results were observed for toxin B, with >99% amino acid sequence identity among strains within toxinotypes 0, III, IV, and VIII. Phylogenetic analysis showed that the protein sequences for toxins A and B clustered with already-published sequences in a toxinotype-specific manner, indicating that the isolates analyzed harbored representative toxin A and B sequences of the known toxinotypes (data not shown). Amino acid sequence comparisons between strains of different toxinotypes and the reference toxinotype 0 strain, VPI 10463, revealed that there was a high degree of sequence conservation between the two toxin types across toxinotypes 0, I, and XII (Table 2). However, the degree of sequence conservation was lower for toxin B from toxinotypes III, V, and VI. Notably, genomic sequencing of toxinotype VIII strains confirmed deletion of the toxin A gene and revealed lower sequence identity for toxin B compared to VPI 10463 than was seen with toxinotype 0 and XII strains.

**TABLE 2:** Toxin sequence identity to the reference strain VPI 10463

Toxinotype	% toxin sequence identity to strain VPI 10463	
	TcdA	TcdB
0	99.8	99.9
III	98.1	92.2
IV	98.4	98.2
V	98.2	96.1
VIII		93.7
I	ND^*a*^	99.9
VI	ND	96.1
XII	99.7	99.8

a ND, not determined.

### Cytotoxic activity of secreted toxins.

**FIG 1:**
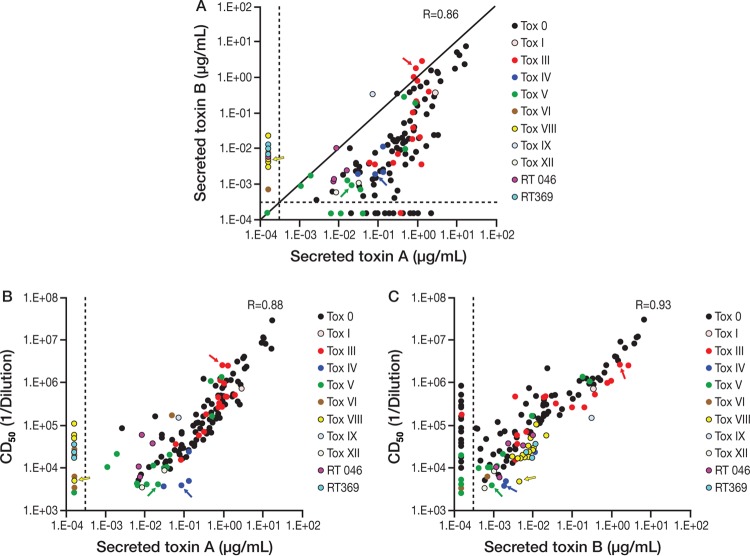
Toxin production and cytotoxicity of clinical isolates from different toxinotypes. Concentrations of secreted toxin A and B in the bacterial supernatant of clinical isolates were quantified by ELISA. (A) Toxicity of the bacterial supernatant was evaluated on IMR-90 cells in a toxin (Tox) cross-neutralization assay, and CD_50_ dilution values were calculated as described in Materials and Methods. (B and C) CD_50_ dilution against toxin A concentration (B) or toxin B concentration (C). Each data point represents a value from an individual clinical isolate. Data points are grouped by toxinotypes. Values below the lower limit of quantification, indicated by the vertical dotted lines, were replaced by values corresponding to half of the limit of quantification. Prototype strains used in the hamster challenge studies are identified by arrows.

Secretion of both toxins A and B was detected in culture supernatant for the majority of C. difficile isolates assessed, with a good correlation between the concentrations of detected toxins A and B (*R* = 0.86) and with more toxin A than toxin B secreted (Fig. 1A). As expected, toxinotype VIII strains produced high levels of toxin B but no toxin A (27, 28). The levels of toxins secreted differed greatly between the different isolates, even among those of the same toxinotype. Overall, no distinct pattern of toxin production could be established for the different toxinotypes, although that might have been related to the capacity of each strain to grow and/or secrete toxins under the culture conditions used (conditions were not optimized for any isolate).

The levels of cytotoxic activity of the secreted toxins also differed among the strains; there was a wide range in the values of the concentrations of toxins inducing 50% cytotoxicity (CD_50_) for culture supernatants across toxinotypes (Fig. 1B and C). Cytotoxicity was correlated with the concentration of toxins A and B in the culture supernatants (correlation coefficients of 0.88 and 0.93, respectively).

### Cross-neutralization of clinical isolates from different toxinotypes.

**FIG 2:**
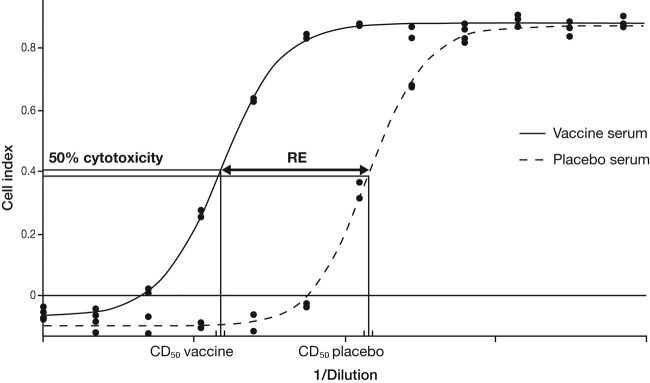
Determination of cytotoxic activity (CD_50_) and neutralization capacity. Determinations of CD_50_ and relative efficacy (RE) values are represented for the bacterial supernatant from the ATCC 43255 strain, corresponding to the homologous vaccine strain. Duplicated serial dilutions of preparations containing toxins were preincubated with sera from hamsters injected with the vaccine or placebo before transfer onto the IM90 cells. The remaining toxic effect on cells was measured by cell index analysis. Each of the data points corresponds to vaccine and placebo cell indices and was plotted according to log_10_-transformed reciprocal dilutions. The 50% cytotoxic dose (CD_50_) was calculated from the four-parameter logistic regression as described in Materials and Methods. The shift between vaccine and placebo cell index curves is defined as the RE. The RE represents the capacity of vaccine-specific antitoxin antibodies to neutralize the toxin-cytotoxic activity.

**FIG 3:**
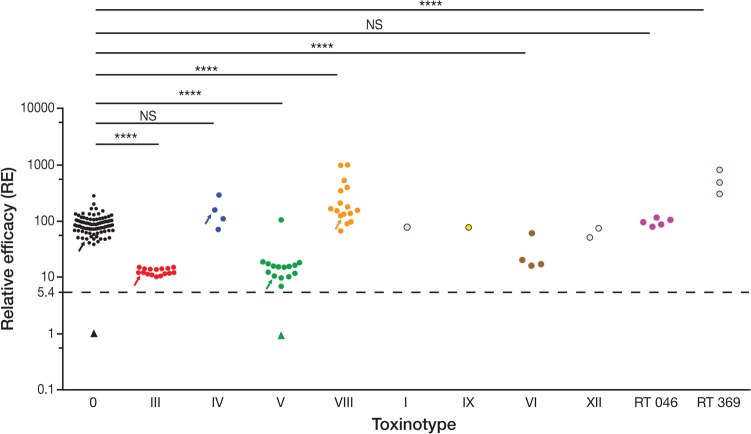
Cross-neutralization of clinical isolates from different toxinotypes. Bacterial supernatants from recent clinical isolates were evaluated for their toxic potency. The relative efficacy (RE) levels of vaccine-specific antitoxin antibodies with respect to neutralization of the cytotoxic activity of secreted toxins from clinical isolates were calculated as described in Materials and Methods. Each data point represents a value from an individual clinical isolate. Data points are grouped by toxinotypes. The dotted line indicates the threshold of statistically significant RE. Significant differences (*P* ≤ 0.0001) are indicated by four asterisks (****). Nonsignificant differences are indicated by “NS.” Prototype strains used in the hamster challenge studies are identified by arrows.

The neutralizing capacity or relative efficacy (RE) (the two terms are used interchangeably) of vaccine-generated antibodies was determined by comparing the CD_50_ values determined for each individual bacterial culture supernatant in the presence of either sera from vaccine-immunized hamsters or sera from placebo-immunized hamsters (see Materials and Methods). This is illustrated for the bacterial supernatant from the ATCC 43255 strain, which corresponds to the homologous vaccine strain (Fig. 2). Pooled sera from 12 hamsters vaccinated with the C. difficile toxoid vaccine neutralized the cytotoxic activity of culture supernatants from the 165 strains assessed—the calculated RE values were all above the statistical threshold (Fig. 3). Interestingly, the neutralizing activities of serum antibodies were similar against culture supernatants from isolates of the same toxinotype, despite the broad cytotoxicity range among isolates. For toxinotypes III, V, and VI, the neutralization capacity was above the threshold but was significantly lower (*P* value < 0.00001 Dunnett's test) than that determined for toxinotype 0 (Fig. 3). Whether the lower neutralization capacity was related to binary toxin activity also produced by these toxinotypes could not be determined, as the IMR90 cells used in the assay were not sufficiently sensitive to purified binary toxin (data not shown). However, a high neutralizing capacity was observed against toxinotype IV or VIII isolates, which are also binary toxin positive (i.e., either not statistically significantly different from the mean RE of toxinotype 0 or with a significantly higher result with a *P* value of <0.00001 [Dunnett's test], respectively). For toxinotype VIII and RT 369, the neutralization capacity was significantly higher than for toxinotype 0 (*P* value, <0.00001 [Dunnett's test]). For the other toxinotypes assessed, the neutralizing capacity was comparable with that determined for toxinotype 0.

### Cross-neutralization of purified toxins from different toxinotypes.

The levels of cytotoxic activity of purified A toxins from the prevalent C. difficile toxinotypes assessed ranged from 4.4 to 15.9 ng/ml (Table 3); in contrast, there was a 10,000-fold difference in the levels of cytotoxic activity (0.2 ng/ml to 6.2 μg/ml) of purified toxin B. Sera from vaccinated hamsters were able to neutralize all purified A and B toxins assessed (RE, >5.4). Interestingly, the range of neutralizing activities against B toxins was broader than for A toxins, consistent with the variations in cytotoxic activity observed for the toxins. These results corroborate those observed with toxins secreted in culture supernatants from the different isolates and suggest an important role for vaccine-induced antibodies in protecting against the cytotoxic activities of toxins A and B from different toxinotypes.

**TABLE 3:** Cytotoxic activity of purified toxins from prevalent C. difficile toxinotypes and relative efficacy of neutralization by sera from vaccine-immunized hamsters

Toxinotype	PCR ribotype	Value for purified native toxin^*a*^:			
		A		B	
		CD_50_ (ng/ml)	RE	CD_50_ (ng/ml)	RE
0	087	4.4	52.3	0.2	38.1
	001	15.9	24.4	108.0	818.5
	002	5.8	44.4	98.1	387.4
	014	8.9	68.3	6,220.0	95.7
III	027	5.4	17.8	3.5	9.3
IV	023	9.4	15.4	ND	ND
V	078	6.9	14.1	4.5	13.1
VIII	017	ND	ND	17.1	149.7

a CD_50_, concentration of toxin inducing 50% cytotoxicity; RE, relative efficacy of neutralization, considered statistically significant if above the threshold value of 5.4; ND, not determined. Data represent geometric means of results from 3 independent experiments.

### Vaccine-induced cross-protection against prototype strains in the hamster challenge model.

Since sera from vaccinated hamsters exhibited a homogeneous neutralization capacity (RE) for isolates of the same toxin variant type *in vitro*, only one prototype strain was selected as representative of each prevalent toxinotype (Table 1) in the hamster lethal infection challenge model. The vaccine provided significant protection against lethal infection and disease symptoms caused by the five most prevalent toxinotypes (Table 4; see also Fig. S1 in the supplemental material), encompassing toxin-producing phenotypes A+B+CDT–, A–B+CDT–, and A+B+CDT+.

**TABLE 4:** C. difficile hamster challenge model using five of the most prevalent representative toxin variant strains

Challenge prototype strain, toxin production profile (spore inoculum used for challenge)	Result^*a*^				Between-group statistical analyses^*b*^
	Placebo		C. difficile toxoid vaccine		
	Observations (symptoms)	Lethality rate	Observations (symptoms)	Lethality rate	
Toxinotype 0 strain 630 (RT 012), A+B+CDT− (3,700 CFU)	Onset of diarrhea (score 1) after 3 days with severe diarrhea (score 3) within 8 days after challenge	8/9 (89) by 13 days	Very limited change in feces (score 1, loose stool)	0/9 (0)	Symptoms, *P* < 0.0001 (Wilcoxon two-sample test); lethality, *P* = 0.0004 (Fisher exact test), *P* = 0.0001 (Kaplan-Meier log rank test)
Toxinotype III strain IPP40348 (RT 027), A+B+CDT+ (5,000 CFU)	Onset of diarrhea (score 1) after 2 days with severe diarrhea (score 3) within 3 days after challenge	12/12 (100) by 4 days	2 with moderate diarrhea (score 2; wet tail and perianal region) within 3 days that died 6 days after challenge	2/12 (17) by 2 days	Symptoms, *P* < 0.0001 (Wilcoxon two-sample test); lethality, *P* < 0.001 (Fisher exact test), *P* ≤ 0.025 (Kaplan-Meier log rank test)
Toxinotype IV strain NK91 (RT 023), A+B+CDT+ (1,200 CFU)	Onset of diarrhea (score 1) within 1 day with severe and acute diarrhea (score 3) within 3 days after challenge	12/12 (100) by 6 days	Mild and transient diarrhea (score 1) which resolved within 13 days	0/12 (0)	Symptoms, *P* < 0.0001 (Wilcoxon two-sample test); lethality, *P* < 0.001 (Fisher exact test), *P* < 0.001 (Kaplan-Meier log rank test)
Toxinotype V strain BAA-1875 (RT 078), A+B+CDT+ (6,380 CFU)	Severe acute diarrhea (score 3) in 50% of hamsters within 2 days with rapid lethality onset	12/12 (100) by 3 days	No disease symptoms	0/12 (0)	Symptoms, *P* < 0.0001 (Wilcoxon two-sample test); lethality, *P* ≤ 0.0001 (Fisher exact test), *P* ≤ 0.0001 (Kaplan-Meier log rank test)
Toxinotype VIII strain ATCC 43598 (RT 017), A−B+CDT− (9,400 CFU)	Severe diarrhea (score 3) within 3 to 13 days after challenge	7/12 (58) within 10 days	No disease symptoms	0/12 (0)	Symptoms, *P* < 0.0001 (Wilcoxon two-sample test); lethality, *P* = 0.0046 (Fisher exact test), *P* = 0.0020 (Kaplan-Meier log rank test)

a For observations (symptom) data, diarrheal disease was reported as a group median score representing individual illness scores defined as follows: 0, no disease; 1, loose feces; 2, wet tail and perianal region; 3, wet perianal region, belly, and hind paws; 4, death. For lethality rate data, values represent the number of hamsters that died/total number of hamsters assessed (percent total deaths).

b The area under the curve of the diarrheal disease scores over time was calculated for each animal. The effect on diarrheal disease symptoms (vaccine versus placebo) was analyzed using an exact Wilcoxon two-sample test. Protection efficacy was assessed as the difference between the survival kinetic percentages (Kaplan-Meier log rank test) and as the difference between the survival percentages 17 days after challenge (Fisher exact test). Both statistical tests were performed with a margin of error of 5%.

## DISCUSSION

The evolving genetic diversity of C. difficile, its worldwide distribution, and the emergence of hypervirulent strains have implications for disease control and make vaccine development more challenging, given the importance of ensuring broad protection against all prevalent strains (11–21). Despite the existence of at least 12 different toxinotypes, extensive genetic analysis of C. difficile indicates that there is a high degree of sequence conservation between exotoxins A and B, the major bacterial virulence factors, which suggests that a vaccine composed of these toxoids could provide broad protection against circulating virulent strains. Preclinical studies performed with the C. difficile toxoid vaccine in the hamster model have shown that the vaccine elicits systemic toxin A- and B-neutralizing antibodies and provides protection against C. difficile lethal challenge with a number of toxinotype strains (23). We have significantly expanded on this body of work by investigating the capacity of sera from vaccinated hamsters to neutralize toxins from a wide variety of C. difficile toxinotypes, as well as the efficacy of the vaccine in the hamster model to protect against the most prevalent toxinotypes worldwide.

The pattern of toxin A and B secretion from our large collection of C. difficile toxin variants under anaerobic culture conditions was broadly as expected for all strains, i.e., was dependent on the toxinotype. Although the stoichiometries of toxin A and B secretion differed greatly among strains and toxinotypes, toxin A levels were consistently higher than toxin B levels. In addition, the cytotoxic activities of secreted toxins differed greatly but were nonetheless correlated closely with the concentrations of toxin A and B detected, suggesting that the secreted toxins were largely biologically active. Sera from hamsters vaccinated with the vaccine consistently neutralized the cytotoxic activity of culture supernatants from all 165 strains evaluated, albeit with variable levels of relative efficacy for strains of different toxinotypes. In contrast, the neutralizing capacities of the sera were relatively uniform for isolates of the same toxinotype. These results might be linked to differences in the affinity of vaccine-specific antibodies for particular toxin types, as seroneutralization was measured under conditions where the toxin-specific antibody concentration was not limiting relative to the concentration of toxin. Studies performed with monoclonal antibodies (MAbs) have shown that neutralization potency correlates with antibody-toxin binding affinities and that they were protective against a broad range of genetically diverse C. difficile strains (29–32).

We found a larger disparity in the levels of cytotoxic activity of purified toxin B from prevalent C. difficile toxinotypes than in those seen with purified toxin A. This disparity may have been associated with the differential affinities of the toxin variants for the toxin receptor in IMR-90 cells or with other differences in the endocytic pathways used during the intoxication process (33). Of note, although toxin B appears to be more cytotoxic than toxin A in cell culture (34), the latter is more enterotoxic in animal oral intoxication models (35). Nonetheless, purified toxins A and B from all isolates assessed were effectively neutralized by sera from vaccinated hamsters, confirming the broad neutralizing capacity observed with culture supernatants. Overall, cross-neutralization was shown for purified toxins A and B or culture supernatants from all prevalent toxinotypes (0, III, IV, V, and VIII), as well as for culture supernatants of the other commonly circulating toxinotypes worldwide (I, VI, IX, and XII). Of note, the neutralization assay was highly sensitive in evaluating C. difficile cytotoxicity, which enabled us to demonstrate homogeneous neutralization capacities among several isolates from the same toxinotype and thus to select representative prototypes from the five most prevalent toxinotypes for the *in vivo* studies.

The C. difficile toxoid vaccine conferred significant protection against disease symptoms and death caused by representative prototype strains from the most prevalent toxinotypes in the hamster challenge model. The protection observed against the toxinotype III/RT 027 challenge is of particular interest, as these strains are hypervirulent and are known to produce more toxin than the reference strain. Although results of an earlier study had suggested that a vaccine containing toxin A and B as well as binary toxin improved efficacy against challenge with toxinotype III/RT 027 compared with the bivalent vaccine (36), our candidate vaccine nonetheless provided protection against strains that additionally expressed binary toxin (toxinotypes III, IV, and V). The difference in vaccine effectiveness could be explained by differences in toxin production. Our vaccine candidate contains native toxins detoxified with formalin, whereas the vaccine tested in the earlier study contained recombinant molecularly detoxified toxins (5 point mutations) that had been produced in a baculovirus expression system and then additionally detoxified with formalin. In addition, differences in the way the hamster model was conducted, such as in clindamycin pretreatment, the strain used for challenge, and spore preparation, could have had an impact on the effectiveness of the vaccine candidate. In accordance with our approach, other vaccines using recombinant fragments of toxins A and B were shown to protect against challenge with toxinotype III/RT 027 strains in the hamster challenge model (37, 38). In one study, ovine toxin A/toxin B antibodies were demonstrated to be effective against challenge with prototype strains of toxinotypes 0, III, and V in the hamster challenge model (39). In humans, targeting of both toxins A and B with humanized MAbs actoxumab and bezlotoxumab, respectively, was effective in protecting against CDI recurrence, including infection with the epidemic 027 strain (40). These results are encouraging, as numerous studies have suggested a correlation between binary toxin-positive strains and increased mortality rates in CDI patients, but it remains unclear whether the binary toxin is responsible for increased disease severity (10). CDT induces formation of microtubule-based protrusion and increases adherence of bacteria to intestinal epithelium (41, 42); A–B–CDT+ strains, including toxinotype XI/RT 033 or RT 033-like strains, have been isolated only sporadically (43). The vaccine also protected against challenge with strains of toxinotype VIII/RT 017, phenotype A–B+CDT–, emphasizing the important role of toxin B neutralization in protection and consistent with observations corresponding to the use of bezlotoxumab against recurrent CDI in a recent clinical study (44).

Our study results show that sera from hamsters vaccinated with the candidate C. difficile toxoid vaccine neutralized the cytotoxic activity of secreted and purified C. difficile variant toxins A and B and conferred protection against disease symptoms and death in the hamster model against a broad range of clinically relevant toxinotype strains. In humans, the vaccine candidate has also been found to generate a strong toxin-neutralizing response (as demonstrated in phase I and II studies in human volunteers [24–26]). However, recent observations in a phase III clinical study in older adult volunteers (≥50 years of age) failed to provide evidence of its protective efficacy, and subsequent development of the candidate vaccine has ceased (45). Additional research is therefore needed to determine the predictive value, if any, of the hamster model described here (i.e., acute primary infection of young animals) with regard to vaccine-mediated protection in a natural infection setting, as well as with regard to the role of toxin-neutralizing antibodies in protection against CDI in humans.

## MATERIALS AND METHODS

### C. difficile clinical isolates and prototype strains.

The 165 C. difficile clinical isolates and prototype strains used in the neutralization assessments are summarized in Table 1; origins and sources are described in the supplemental material.

### Genome sequencing and phylogenetic analysis.

Whole-genome sequencing of 49 strains (Table 1) was performed using Illumina HiSeq, MiSeq, and NextSeq platforms (Illumina San Diego, CA), generating 2 × 100-bp, 2 × 300-bp, and 2 × 150-bp paired-end reads, respectively. Sequence reads were trimmed and assembled *de novo* with CLC Genomics Workbench 8.5 (Qiagen Bioinformatics, Hilden, Germany). Toxin A and toxin B gene sequences were extracted from the *de novo* contigs and translated. Published protein sequences were retrieved from Uniprot database (http://www.uniprot.org/). Multiple-sequence alignment was performed with the MUSCLE algorithm (46). Pairwise sequence comparison was performed with CLC Genomics Workbench 8.5. The phylogenetic tree was constructed with the FastTree algorithm (47) and visualized with Dendroscope (48).

### Secreted toxins from bacterial culture supernatants.

C. difficile isolates and prototype strains were grown anaerobically in soy-yeast extract-salt (SYS) medium for 16 h and then in SYS medium supplemented with sorbitol for 72 h. Bacterial culture supernatants were harvested by filtration through 0.22-μm-pore-size filters, supplemented with anti-proteases and 30% glycerol, and stored at −80°C until analysis. Quantitation of toxins A and B present in the supernatants was performed using a commercial enzyme-linked immunosorbent assay (ELISA) method (tgcBIOMICS GmbH, Bingen, Germany) according to the manufacturer's instructions. The detection limit was 0.31 ng/ml.

### Purified toxins A and B.

Purified toxins A and B from strain VPI 10463 (toxinotype 0/RT 087) were prepared as described elsewhere (49). Purified toxins A and B from toxinotype 0/RT 001, 002, 014, and 106; toxinotype III/RT 027; toxinotype IV/RT 023; and toxinotype V/RT 078 and purified toxin B from toxinotype VIII/RT 017 were purchased from tgcBIOMICS GmbH (Bingen, Germany).

### Cell-based cytotoxicity and cross-neutralization assays.

The cytotoxicity activities of secreted or purified toxins and the neutralization capacities of serum antibodies were assessed on IMR-90 cells by monitoring changes in cellular electrical impedance using the RCTA xCELLigence system (ACEA Biosciences Inc., Ozyme, Saint-Quentin-en-Yvelines, France) as described elsewhere (34). Sera used for *in vitro* cross-neutralization assay were either pooled vaccine sera or pooled placebo sera from 12 hamsters that received three intramuscular injections of either vaccine at a dose of 5 μg toxoid (1/20 of the human dose) in the presence of aluminum hydroxide or placebo, respectively. Serum raised against the vaccine was known to neutralize cytotoxic activity of purified toxins of bacterial supernatant from the vaccine strain. Briefly, a predetermined dilution (1/400) of either hamster placebo serum or serum raised against the vaccine was preincubated for 60 min with serial dilutions of secreted toxins from the isolates or purified toxin A or toxin B. The selection of the serum dilution was based on results of testing performed with serial dilutions of bacterial supernatant from the vaccine homologous strain to obtain sigmoidal dose-response curves and a resultant shift to the right in the sigmoid dose-response curves from comparisons of vaccine serum to placebo serum. The serum-toxin mixture was then added onto IMR-90 cells, which were preseeded onto E-Plates (ACEA) to reach confluence and attachment on the electrodes. Plates were then incubated at 37°C for 21 h before cellular electrical impedance assessment; rounding of IMR90 cells induced by active toxin led to a decrease in electrode impedance, displayed as cell index values.

### C. difficile hamster model. (i) Ethical use of animals.

All animal experiments were performed in compliance with European Directive 2010/63 and national regulations. Studies were conducted in animal facilities accredited by the Association for Assessment and Accreditation of Laboratory Animal Care International. The protocols were approved by the committee on the ethics of animal experiments at Sanofi Pasteur, France, and all efforts were made to reduce the use of animals and to minimize pain and distress.

### (ii) Animals.

Female Golden Syrian hamsters (Mesocricetus auratus; Charles River Laboratories, Germany) (70 to 90 g) were used for immunization and challenge studies. The hamsters were randomly distributed within groups and housed 3 per 800-cm^2^ cage (Serlab); they were housed individually with isocaps after C. difficile challenge.

### (iii) C. difficile toxoid vaccine and placebo.

The C. difficile toxoid vaccine (highly purified toxins A and B from C. difficile reference strain VPI 10463) was presented as a freeze-dried preparation that was reconstituted with diluent and mixed with aluminum hydroxide adjuvant as described elsewhere (49). Aluminum hydroxide adjuvant in diluent buffer was used as the placebo control.

### (iv) C. difficile spore-enriched preparation for challenge.

C. difficile strains were anaerobically grown in thioglycolate medium for 24 h at 37°C. The culture was then inoculated onto anaerobic blood agar plates (CDC, Becton Dickinson, Sparks, MD, USA) and incubated at 37°C for 7 days to induce spore formation. Spores were then harvested into phosphate-buffered saline (PBS) without Ca^2+^ or Mg^2+^, washed twice, and then heat shocked at 57°C for 10 min to kill the vegetative cells. The spore suspension was centrifuged at 500 × *g* for 30 min, resuspended in 20% glycerol–phosphate-buffered saline (PBS), and then frozen at below −70°C for long-term storage. Viable spore counts were assessed by performing serial 10-fold dilutions in water, and the reaction mixtures were plated in triplicate onto brain heart infusion medium (with yeast extract) agar plates (Becton Dickinson) in the presence of 0.1% of taurocholate (Sigma, Saint-Quentin-Fallavier, France) to enhance spore recovery. Plates were incubated under anaerobic conditions at 37°C for at least 48 h before the colonies were quantified as CFU counts per milliliter.

### (v) C. difficile vaccination and challenge.

The hamsters (9 to 12 per group) received three intramuscular injections of either vaccine (at a dose of 5 μg toxoid [1/20 of the human dose] in the presence of aluminum hydroxide) or placebo 2 weeks apart. On day 35, sera were collected for seroneutralization assessment. On day 41, clindamycin-2-phosphate antibiotic solution (Sigma-Aldrich) (10 mg/kg of body weight) was administered intraperitoneally to disrupt the gut microbiota and to render the animals susceptible to subsequent lethal challenge. After 24 h, the hamsters were challenged intragastrically, using a feeding needle, with a predetermined lethal dose of a live C. difficile spore-enriched preparation (prepared as described above) of each of the selected prototype strains (Table 1). The animals were monitored postchallenge at least twice daily for morbidity and mortality. Body weight was also monitored prior to the clindamycin injection and then 1 to 3 times per week for the study duration.

### Statistical analysis. (i) Determination of cytotoxic activity.

Duplicated serial dilutions of bacterial supernatant containing secreted toxins from each clinical isolate or purified toxin were preincubated with pooled sera from 12 hamsters injected with either the vaccine or placebo (1/400), respectively, before transfer onto the IM90 cells. Corresponding vaccine and placebo cell indices were plotted according to the log_10_-transformed reciprocal dilutions and modeled using a four-parameter logistic regression and in-house software as follows:

(1) $${\text{cell}\;\;\text{index}}_{i}=A+\frac{B-A}{\left({1+10}^{\left\{-\text{slope}+[\text{log}\;\left(1/{\text{dilution}}_{i}\right)-\text{log}\;C]\right\}}\right)}$$

where *A* = lower asymptote and *B* = higher asymptote. The *C* parameter of the logistic regression corresponds to the reciprocal dilution of the bacterial supernatant or the concentration of purified toxins that induced 50% of the maximum cell index, defined as the 50% cytotoxic dose (CD_50_) (Fig. 2).

### (ii) Determination of seroneutralization capacity.

The shift between the vaccine and placebo cell index curves was defined as the relative efficacy (RE) and calculated as the ratio between vaccine CD_50_ values and placebo CD_50_ values as follows:

(2) $$\text{RE}=\frac{{\text{CD}}_{50}\;\text{placebo}}{{\text{CD}}_{50}\;\text{vaccine}}$$

RE represents the capacity of vaccine-specific antitoxin antibodies to neutralize the toxin-cytotoxic activity either from purified toxins or from supernatant from clinical isolate cultures (Fig. 2). The threshold value for the RE level considered statistically significant was established by the determination of 95% intermediate precision confidence intervals, using either specific antitoxin antibodies or irrelevant antibodies against placebo, and was defined as 5.4.

The mean RE obtained with the different toxinotypes from isolates was compared to that determined for toxinotype 0 using a pairwise two-way analysis of variance (ANOVA) with a Dunnett adjustment at an alpha level of 5% in GraphPad Prism v 6.7 (GraphPad Software, Inc., San Diego, California). All data were log_10_ transformed to normalize distribution values prior to statistical analyses. Data from toxinotypes I, IX, and XII were not taken into account in these analyses due to the low number of strains per group.

## Supplementary Material

Supplemental material

## References

[1] Kelly CP, Pothoulakis C, LaMont JT 1994 Clostridium difficile colitis. N Engl J Med 330:257–262. doi:10.1056/NEJM199401273300406. 8043060

[2] Hall AJ, Curns AT, McDonald LC, Parashar UD, Lopman BA 2012 The roles of Clostridium difficile and norovirus among gastroenteritis-associated deaths in the United States, 1999–2007. Clin Infect Dis 55:216–223. doi:10.1093/cid/cis386. 22491338

[3] Ghose C, Kelly CP 2015 The prospect for vaccines to prevent Clostridium difficile infection. Infect Dis Clin North Am 29:145–162. doi:10.1016/j.idc.2014.11.013. 25677708

[4] Leffler DA, Lamont JT 2015 Clostridium difficile infection. N Engl J Med 373:287–288. doi:10.1056/NEJMc1506004. 26176396

[5] Shen A 2012 Clostridium difficile toxins: mediators of inflammation. J Innate Immun 4:149–158. doi:10.1159/000332946. 22237401 PMC3388264

[6] Sun X, Savidge T, Feng H 2010 The enterotoxicity of Clostridium difficile toxins. Toxins 2:1848–1880. doi:10.3390/toxins2071848. 22069662 PMC3153265

[7] Voth DE, Ballard JD 2005 Clostridium difficile toxins: mechanism of action and role in disease. Clin Microbiol Rev 18:247–263. doi:10.1128/CMR.18.2.247-263.2005. 15831824 PMC1082799

[8] Rupnik M 2008 Heterogeneity of large clostridial toxins: importance of Clostridium difficile toxinotypes. FEMS Microbiol Rev 32:541–555. doi:10.1111/j.1574-6976.2008.00110.x. 18397287

[9] Rupnik M, Janezic S 2016 An update on Clostridium difficile toxinotyping. J Clin Microbiol 54:13–18. doi:10.1128/JCM.02083-15. 26511734 PMC4702747

[10] Gerding DN, Johnson S, Rupnik M, Aktories K 2014 Clostridium difficile binary toxin CDT: mechanism, epidemiology, and potential clinical importance. Gut Microbes 5:15–27. doi:10.4161/gmic.26854. 24253566 PMC4049931

[11] Barbut F, Mastrantonio P, Delmee M, Brazier J, Kuijper E, Poxton I 2007 Prospective study of Clostridium difficile infections in Europe with phenotypic and genotypic characterisation of the isolates. Clin Microbiol Infect 13:1048–1057. doi:10.1111/j.1469-0691.2007.01824.x. 17850341

[12] Bauer MP, Notermans DW, van Benthem BH, Brazier JS, Wilcox MH, Rupnik M, Monnet DL, van Dissel JT, Kuijper EJ 2011 Clostridium difficile infection in Europe: a hospital-based survey. Lancet 377:63–73. doi:10.1016/S0140-6736(10)61266-4. 21084111

[13] Cheknis AK, Sambol SP, Davidson DM, Nagaro KJ, Mancini MC, Hidalgo-Arroyo GA, Brazier JS, Johnson S, Gerding DN 2009 Distribution of Clostridium difficile strains from a North American, European and Australian trial of treatment for *C. difficile* infections: 2005–2007. Anaerobe 15:230–233. doi:10.1016/j.anaerobe.2009.09.001. 19737618

[14] Dingle KE, Griffiths D, Didelot X, Evans J, Vaughan A, Kachrimanidou M, Stoesser N, Jolley KA, Golubchik T, Harding RM, Peto TE, Fawley W, Walker AS, Wilcox M, Crook DW 2011 Clinical Clostridium difficile: clonality and pathogenicity locus diversity. PLoS One 6:e19993. doi:10.1371/journal.pone.0019993. 21625511 PMC3098275

[15] Miller M, Gravel D, Mulvey M, Taylor G, Boyd D, Simor A, Gardam M, McGeer A, Hutchinson J, Moore D, Kelly S 2010 Health care-associated Clostridium difficile infection in Canada: patient age and infecting strain type are highly predictive of severe outcome and mortality. Clin Infect Dis 50:194–201. doi:10.1086/649213. 20025526

[16] Stabler RA, Dawson LF, Valiente E, Cairns MD, Martin MJ, Donahue EH, Riley TV, Songer JG, Kuijper EJ, Dingle KE, Wren BW 2012 Macro and micro diversity of Clostridium difficile isolates from diverse sources and geographical locations. PLoS One 7:e31559. doi:10.1371/journal.pone.0031559. 22396735 PMC3292544

[17] Balassiano IT, Yates EA, Domingues RM, Ferreira EO 2012 Clostridium difficile: a problem of concern in developed countries and still a mystery in Latin America. J Med Microbiol 61:169–179. doi:10.1099/jmm.0.037077-0. 22116982

[18] Collins DA, Hawkey PM, Riley TV 2013 Epidemiology of Clostridium difficile infection in Asia. Antimicrob Resist Infect Control 2:21. doi:10.1186/2047-2994-2-21. 23816346 PMC3718645

[19] Freeman J, Bauer MP, Baines SD, Corver J, Fawley WN, Goorhuis B, Kuijper EJ, Wilcox MH 2010 The changing epidemiology of Clostridium difficile infections. Clin Microbiol Rev 23:529–549. doi:10.1128/CMR.00082-09. 20610822 PMC2901659

[20] Cairns MD, Stabler RA, Shetty N, Wren BW 2012 The continually evolving Clostridium difficile species. Future Microbiol 7:945–957. doi:10.2217/fmb.12.73. 22913354

[21] Elliott B, Androga GO, Knight DR, Riley TV 2017 Clostridium difficile infection: evolution, phylogeny and molecular epidemiology. Infect Genet Evol 49:1–11. doi:10.1016/j.meegid.2016.12.018. 28012982

[22] Giannasca PJ, Warny M 2004 Active and passive immunization against Clostridium difficile diarrhea and colitis. Vaccine 22:848–856. doi:10.1016/j.vaccine.2003.11.030. 15040937

[23] Anosova NG, Brown AM, Li L, Liu N, Cole LE, Zhang J, Mehta H, Kleanthous H 2013 Systemic antibody responses induced by a two-component Clostridium difficile toxoid vaccine protect against *C. difficile*-associated disease in hamsters. J Med Microbiol 62:1394–1404. doi:10.1099/jmm.0.056796-0. 23518659

[24] Foglia G, Shah S, Luxemburger C, Pietrobon PJ 2012 Clostridium difficile: development of a novel candidate vaccine. Vaccine 30:4307–4309. doi:10.1016/j.vaccine.2012.01.056. 22682287

[25] Greenberg RN, Marbury TC, Foglia G, Warny M 2012 Phase I dose finding studies of an adjuvanted Clostridium difficile toxoid vaccine. Vaccine 30:2245–2249. doi:10.1016/j.vaccine.2012.01.065. 22306375

[26] de Bruyn G, Saleh J, Workman D, Pollak R, Elinoff V, Fraser NJ, Lefebvre G, Martens M, Mills RE, Nathan R, Trevino M, van Cleeff M, Foglia G, Ozol-Godfrey A, Patel DM, Pietrobon PJ, Gesser R 2016 Defining the optimal formulation and schedule of a candidate toxoid vaccine against Clostridium difficile infection: a randomized phase 2 clinical trial. Vaccine 34:2170–2178. doi:10.1016/j.vaccine.2016.03.028. 27013431

[27] Borriello SP, Wren BW, Hyde S, Seddon SV, Sibbons P, Krishna MM, Tabaqchali S, Manek S, Price AB 1992 Molecular, immunological, and biological characterization of a toxin A-negative, toxin B-positive strain of Clostridium difficile. Infect Immun 60:4192–4199. 1398930 10.1128/iai.60.10.4192-4199.1992 PMC257452

[28] Kato H, Ito Y, Akahane T, Izumida S, Yokoyama T, Kaji C, Arakawa Y 2010 Typing of Clostridium difficile isolates endemic in Japan by sequencing of slpA and its application to direct typing. J Med Microbiol 59:556–562. doi:10.1099/jmm.0.016147-0. 20133413

[29] Anosova NG, Cole LE, Li L, Zhang J, Brown AM, Mundle S, Zhang J, Ray S, Ma F, Garrone P, Bertraminelli N, Kleanthous H, Anderson SF 2015 A combination of three fully human toxin A- and toxin B-specific monoclonal antibodies protects against challenge with highly virulent epidemic strains of Clostridium difficile in the hamster model. Clin Vaccine Immunol 22:711–725. doi:10.1128/CVI.00763-14. 25924765 PMC4478530

[30] Hernandez LD, Racine F, Xiao L, DiNunzio E, Hairston N, Sheth PR, Murgolo NJ, Therien AG 2015 Broad coverage of genetically diverse strains of Clostridium difficile by actoxumab and bezlotoxumab predicted by in vitro neutralization and epitope modeling. Antimicrob Agents Chemother 59:1052–1060. doi:10.1128/AAC.04433-14. 25451052 PMC4335902

[31] Marozsan AJ, Ma D, Nagashima KA, Kennedy BJ, Kang Y, Arrigale RR, Donovan GP, Magargal WW, Maddon PJ, Olson WC 2012 Protection against Clostridium difficile infection with broadly neutralizing antitoxin monoclonal antibodies. J Infect Dis 206:706–713. doi:10.1093/infdis/jis416. 22732923 PMC3491748

[32] Qiu H, Cassan R, Johnstone D, Han X, Joyee AG, McQuoid M, Masi A, Merluza J, Hrehorak B, Reid R, Kennedy K, Tighe B, Rak C, Leonhardt M, Dupas B, Saward L, Berry JD, Nykiforuk CL 2016 Novel Clostridium difficile anti-toxin (TcdA and TcdB) humanized monoclonal antibodies demonstrate in vitro neutralization across a broad spectrum of clinical strains and in vivo potency in a hamster spore challenge model. PLoS One 11:e0157970. doi:10.1371/journal.pone.0157970. 27336843 PMC4919053

[33] Chandrasekaran R, Kenworthy AK, Lacy DB 2016 Clostridium difficile toxin A undergoes clathrin-independent, PACSIN2-dependent endocytosis. PLoS Pathog 12:e1006070. doi:10.1371/journal.ppat.1006070. 27942025 PMC5152916

[34] D'Auria KM, Bloom MJ, Reyes Y, Gray MC, van Opstal EJ, Papin JA, Hewlett EL 2015 High temporal resolution of glucosyltransferase dependent and independent effects of Clostridium difficile toxins across multiple cell types. BMC Microbiol 15:7. doi:10.1186/s12866-015-0361-4. 25648517 PMC4323251

[35] D'Auria KM, Kolling GL, Donato GM, Warren CA, Gray MC, Hewlett EL, Papin JA 2013 In vivo physiological and transcriptional profiling reveals host responses to Clostridium difficile toxin A and toxin B. Infect Immun 81:3814–3824. doi:10.1128/IAI.00869-13. 23897615 PMC3811747

[36] Secore S, Wang S, Doughtry J, Xie J, Miezeiewski M, Rustandi RR, Horton M, Xoconostle R, Wang B, Lancaster C, Kristopeit A, Wang S-C, Christanti S, Vitelli S, Gentile M-P, Goerke A, Skinner J, Strable E, Thiriot DS, Bodmer J-L, Heinrichs JH 2017 Development of a novel vaccine containing binary toxin for the prevention of Clostridium difficile disease with enhanced efficacy against NAP1 strains. PLoS One 12:e0170640. doi:10.1371/journal.pone.0170640. 28125650 PMC5268477

[37] Leuzzi R, Spencer J, Buckley A, Brettoni C, Martinelli M, Tulli L, Marchi S, Luzzi E, Irvine J, Candlish D, Veggi D, Pansegrau W, Fiaschi L, Savino S, Swennen E, Cakici O, Oviedo-Orta E, Giraldi M, Baudner B, D'Urzo N, Maione D, Soriani M, Rappuoli R, Pizza M, Douce GR, Scarselli M 2013 Protective efficacy induced by recombinant Clostridium difficile toxin fragments. Infect Immun 81:2851–2860. doi:10.1128/IAI.01341-12. 23716610 PMC3719595

[38] Spencer J, Leuzzi R, Buckley A, Irvine J, Candlish D, Scarselli M, Douce GR 2014 Vaccination against Clostridium difficile using toxin fragments: observations and analysis in animal models. Gut Microbes 5:225–232. doi:10.4161/gmic.27712. 24637800 PMC4063849

[39] Roberts A, McGlashan J, Al-Abdulla I, Ling R, Denton H, Green S, Coxon R, Landon J, Shone C 2012 Development and evaluation of an ovine antibody-based platform for treatment of Clostridium difficile infection. Infect Immun 80:875–882. doi:10.1128/IAI.05684-11. 22144483 PMC3264293

[40] Lowy I, Molrine DC, Leav BA, Blair BM, Baxter R, Gerding DN, Nichol G, Thomas WD Jr, Leney M, Sloan S, Hay CA, Ambrosino DM 2010 Treatment with monoclonal antibodies against Clostridium difficile toxins. N Engl J Med 362:197–205. doi:10.1056/NEJMoa0907635. 20089970

[41] Schwan C, Kruppke AS, Nölke T, Schumacher L, Koch-Nolte F, Kudryashev M, Stahlberg H, Aktories K 2014 Clostridium difficile toxin CDT hijacks microtubule organization and reroutes vesicle traffic to increase pathogen adherence. Proc Natl Acad Sci U S A 111:2313–2318. doi:10.1073/pnas.1311589111. 24469807 PMC3926047

[42] Schwan C, Stecher B, Tzivelekidis T, van Ham M, Rohde M, Hardt W-D, Wehland J, Aktories K 2009 Clostridium difficile toxin CDT induces formation of microtubule-based protrusions and increases adherence of bacteria. PLoS Pathog 5:e1000626. doi:10.1371/journal.ppat.1000626. 19834554 PMC2757728

[43] Eckert C, Emirian A, Le Monnier A, Cathala L, De Montclos H, Goret J, Berger P, Petit A, De Chevigny A, Jean-Pierre H, Nebbad B, Camiade S, Meckenstock R, Lalande V, Marchandin H, Barbut F 2015 Prevalence and pathogenicity of binary toxin-positive Clostridium difficile strains that do not produce toxins A and B. New Microbes New Infect 3:12–17. doi:10.1016/j.nmni.2014.10.003. 25755885 PMC4337936

[44] Wilcox MH, Gerding DN, Poxton IR, Kelly C, Nathan R, Birch T, Cornely OA, Rahav G, Bouza E, Lee C, Jenkin G, Jensen W, Kim Y-S, Yoshida J, Gabryelski L, Pedley A, Eves K, Tipping R, Guris D, Kartsonis N, Dorr M-B 2017 Bezlotoxumab for prevention of recurrent Clostridium difficile infection. N Engl J Med 376:305–317. doi:10.1056/NEJMoa1602615. 28121498

[45] Sanofi. 2017 Sanofi ends development of Clostridium difficile vaccine. http://mediaroom.sanofi.com/sanofi-ends-development-of-clostridium-difficile-vaccine/ Accessed 12 March 2018.

[46] Edgar RC 2004 MUSCLE: a multiple sequence alignment method with reduced time and space complexity. BMC Bioinformatics 5:113. doi:10.1186/1471-2105-5-113. 15318951 PMC517706

[47] Price MN, Dehal PS, Arkin AP 2010 FastTree 2—approximately maximum-likelihood trees for large alignments. PLoS One 5:e9490. doi:10.1371/journal.pone.0009490. 20224823 PMC2835736

[48] Huson DH, Scornavacca C 2012 Dendroscope 3: an interactive tool for rooted phylogenetic trees and networks. Syst Biol 61:1061–1067. doi:10.1093/sysbio/sys062. 22780991

[49] Sougioultzis S, Kyne L, Drudy D, Keates S, Maroo S, Pothoulakis C, Giannasca PJ, Lee CK, Warny M, Monath TP, Kelly CP 2005 Clostridium difficile toxoid vaccine in recurrent *C. difficile*-associated diarrhea. Gastroenterology 128:764–770. doi:10.1053/j.gastro.2004.11.004. 15765411

