# Supplementary material for: Clostridium difficile Toxoid Vaccine Candidate Confers Broad Protection against a Range of Prevalent Circulating Strains in a Nonclinical Setting
Source: Infect Immun. 2018 May 22;86(6):e00742-17. doi: 10.1128/IAI.00742-17 (PMC5964523; doi:10.1128/IAI.00742-17)
Supplement: Supplemental material [file supp_86_6_e00742-17__index.html]

Supplemental material 

# Clostridium difficile Toxoid Vaccine Candidate Confers Broad Protection against a Range of Prevalent Circulating Strains in a Nonclinical Setting

## Supplemental material

- Supplemental file 1 -

  Supplemental information. Fig. S1. *C. difficile* toxoid vaccine confers cross-protection against challenge with different toxinotype strains.

  PDF, 106K
