# Supplementary material for: Clostridium difficile Toxoid Vaccine Candidate Confers Broad Protection against a Range of Prevalent Circulating Strains in a Nonclinical Setting
Source: Infect Immun. 2018 May 22;86(6):e00742-17. doi: 10.1128/IAI.00742-17 (PMC5964523; doi:10.1128/IAI.00742-17)
Supplement: Supplemental material [file IAI.00742-17_zii999092435s1.pdf]

SUPPLEMENTARY INFORMATION

C. difficile strain collection assessed

The large collection of 165 prevalent circulating *C. difficile* toxin variant strains, including 153 recent clinical isolates, was established from prospective clinical and epidemiological studies worldwide (**Table 1**). The selection was based on multiple criteria such as countries of origin, molecular typing and when available clinical parameters such as *Clostridium difficile* infection (CDI) severity, CDI episode and reflects the current molecular epidemiology (1–11). The collection included recent circulating clinical isolates and a large panel of isolates analyzed for each toxinotype. The geographical and molecular distribution of clinical isolates is described in **Table 1**. Eighty six clinical isolates were collected in fourteen countries throughout Europe (kindly provided by F. Barbut, Saint-Antoine Hospital, Paris, France) (1). Sixteen isolates were also collected in a French hospital between 2010 & 2011 (kindly provided by P. Vanhems Lyon, France) (12). Fifteen clinical isolates were collected in 2011 in the USA and four were collected in Argentina (13) (kindly provided by D. Gerding (Hine VA Hospital, IL, USA). Thirty eight clinical isolates, were collected between 2012 and 2013 in Asia-Pacific countries, including Australia, Singapore, Japan, Korea, Indonesia and Taiwan (kindly provided by ~~by~~ T. Riley, University of West Australia, Australia). Nine different toxinotypes were represented within the different geographical regions, including the 5 most prevalent ones, with the majority being from toxinotype 0 followed by toxinotype III, V, VIII and IV, respectively. Toxinotype I, IX and XII were also represented within the collection. More than 23 ribotypes (RTs) were represented, with more than 13 RTs for the toxinotype 0 (**Table 1**). Interestingly, two other RTs, RT 046 and RT 369, of unknown-toxinotypes, were collected from the Asia-Pacific region. Twelve prototype strains, representing different known toxinotypes, were also included: ATCC®43255™ (VPI

10463), ATCC®1382™ (630), ATCC®BAA-1870™, ATCC®BAA-1875™ and ATCC®43598™ purchased from ATCC (LGC Standards, Molsheim, France), and the NCTC11204, NCTC11209 and NCTC2729 strains from NCTC (Fisher Scientific, Illkirch, France). Strain IPP40348 isolated in France in 2007 was kindly provided by M. Popoff (Pasteur Institute, Paris, France). Strain CDC 13695 strain, isolated in Canada in 2005, and strain CD196, a non-epidemic strain isolated in France in 1985, and strain R20291, isolated in UK in 2006, were obtained from the Center for Disease Control and Prevention (CDC, Washington, USA).

SUPPLEMENTARY REFERENCES

1. Barbut F, Mastrantonio P, Delmee M, Brazier J, Kuijper E, Poxton I. 2007. Prospective study of Clostridium difficile infections in Europe with phenotypic and genotypic characterisation of the isolates. Clin Microbiol Infect 13:1048-57.
2. Bauer MP, Notermans DW, van Benthem BH, Brazier JS, Wilcox MH, Rupnik M, Monnet DL, van Dissel JT, Kuijper EJ. 2011. Clostridium difficile infection in Europe: a hospital-based survey. Lancet 377:63-73.
3. Cheknis AK, Sambol SP, Davidson DM, Nagaro KJ, Mancini MC, Hidalgo-Arroyo GA, Brazier JS, Johnson S, Gerding DN. 2009. Distribution of Clostridium difficile strains from a North American, European and Australian trial of treatment for C. difficile infections: 2005-2007. Anaerobe 15:230-3.
4. Dingle KE, Griffiths D, Didelot X, Evans J, Vaughan A, Kachrimanidou M, Stoesser N, Jolley KA, Golubchik T, Harding RM, Peto TE, Fawley W, Walker AS, Wilcox M, Crook DW. 2011. Clinical Clostridium difficile: clonality and pathogenicity locus diversity. PLoS One 6:e19993.
5. Miller M, Gravel D, Mulvey M, Taylor G, Boyd D, Simor A, Gardam M, McGeer A, Hutchinson J, Moore D, Kelly S. 2010. Health care-associated Clostridium difficile infection in Canada: patient age and infecting strain type are highly predictive of severe outcome and mortality. Clin Infect Dis 50:194-201.
6. Stabler RA, Dawson LF, Valiente E, Cairns MD, Martin MJ, Donahue EH, Riley TV, Songer JG, Kuijper EJ, Dingle KE, Wren BW. 2012. Macro and micro diversity of Clostridium difficile isolates from diverse sources and geographical locations. PLoS One 7:e31559.

7. Balassiano IT, Yates EA, Domingues RM, Ferreira EO. 2012. Clostridium difficile: a problem of concern in developed countries and still a mystery in Latin America. J Med Microbiol 61:169-79.

8. Collins DA, Hawkey PM, Riley TV. 2013. Epidemiology of Clostridium difficile infection in Asia. Antimicrob Resist Infect Control 2:21.

9. Freeman J, Bauer MP, Baines SD, Corver J, Fawley WN, Goorhuis B, Kuijper EJ, Wilcox MH. 2010. The changing epidemiology of Clostridium difficile infections. Clin Microbiol Rev 23:529-49.

10. Cairns MD, Stabler RA, Shetty N, Wren BW. 2012. The continually evolving Clostridium difficile species. Future Microbiol 7:945-57.

11. Elliott B, Androga GO, Knight DR, Riley TV. 2017. Clostridium difficile infection: Evolution, phylogeny and molecular epidemiology. Infection, Genetics and Evolution 49:1-11.

12. Khanafer N, Barbut F, Eckert C, Perraud M, Demont C, Luxemburger C, Vanhems P. 2016. Factors predictive of severe Clostridium difficile infection depend on the definition used. Anaerobe 37:43-8.

13. Lopardo G, Morfin-Otero R, Moran V, II, Noriega F, Zambrano B, Luxemburger C, Foglia G, Rivas EE. 2015. Epidemiology of Clostridium difficile: a hospital-based descriptive study in Argentina and Mexico. Braz J Infect Dis 19:8-14.

Figure S1: ***C. difficile* toxoid vaccine confers cross-protection against challenge with different toxinotype strains.** Hamsters (9 or 12/group) were immunized with either aluminum diluent buffer (○) or with *C. difficile* toxoid vaccine (■) and challenged with a lethal dose of a live *C. difficile* spore-enriched preparation of *C. difficile* strains. The spore inoculum administered for each strains were 3'700 cfu for strain 630 (toxinotype 0); 5'000 cfu for strain IPP40348 (toxinotype III); 1'200 cfu for strain NK91 (toxinotype IV); 6'380 cfu for strain BAA-1875 (toxinotype V) and 9'400 cfu for strain ATCC43598 (toxinotype VIII). Hamsters were then monitored at least twice a day during 17 days post-infection for diarrheal symptoms (A) and survival (B). Diarrheal disease was reported as a group mean score of individual illness scores: 0 – no disease, 1 – loose feces, 2 – wet tail and perianal region, 3 – wet perianal region, belly and hind paws, and 4 – death. Significant difference in feces scoring, calculated by exact Wilcoxon two-sample test, are indicated by **** p-values ≤ 0.0001. Significant differences in survival, calculated by Kaplan Meier log-rank and Fisher exact tests, are indicated by * p-value ≤ 0.1, ** p-value ≤ 0.01, *** p-value ≤ 0.001 *** p-value ≤ 0.0001.

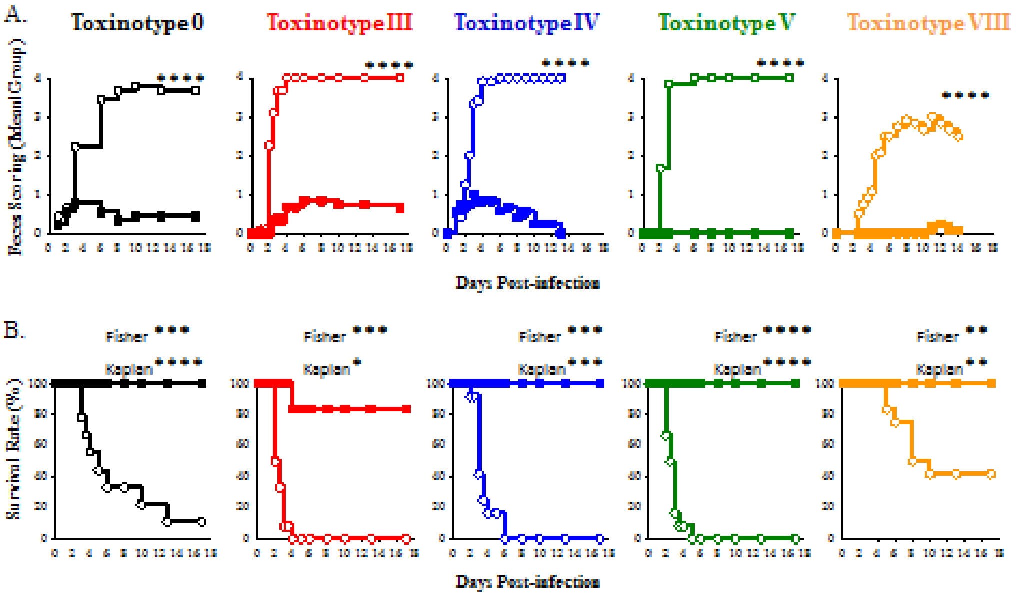

Figure S1, Quemeneur L. et al.
